# Denosumab and breast cancer risk in postmenopausal women: a population-based cohort study

**DOI:** 10.1038/s41416-018-0225-4

**Published:** 2018-11-13

**Authors:** Vasily Giannakeas, Suzanne M. Cadarette, Joann K. Ban, Lorraine Lipscombe, Steven A. Narod, Joanne Kotsopoulos

**Affiliations:** 1 Women’s College Research Institute, Women’s College Hospital, Toronto, ON Canada; 2 Dalla Lana School of Public Health, University of Toronto, Toronto, ON Canada; 3 Institute for Clinical Evaluative Sciences, University of Toronto, Toronto, ON Canada; 4 Leslie Dan Faculty of Pharmacy, University of Toronto, Toronto, ON Canada

**Keywords:** Breast cancer, Risk factors

## Abstract

**Background:**

Denosumab inhibits the receptor activator of nuclear factor κB (RANK) pathway and is used to treat osteoporosis. Emerging evidence suggests RANK-blockade may play a role in mammary tumourigenesis. Thus, we undertook a population-based study of denosumab use and breast cancer risk in a large cohort of postmenopausal women.

**Methods:**

We included women 67+ years with prior bisphosphonate use who filled a first prescription for denosumab. They were matched on age, date, cumulative prior use of and time since last use of a bisphosphonate to women with no history of denosumab. Cox proportional hazards was used to estimate the hazard ratio (HR) of breast cancer with denosumab use.

**Results:**

A total of 100,368 women were included in the analysis with 1271 incident breast cancer events. Denosumab use was associated with a 13% decreased breast cancer risk (HR = 0.87; 95% CI 0.76–1.00). There was no relationship between increasing number of denosumab doses and breast cancer risk (*P*-trend = 0.15).

**Conclusion:**

These findings suggest a potential protective effect of ever denosumab use on breast cancer risk in a cohort of older women previously treated with bisphosphonates.

## Introduction

The drug denosumab is an anti-RANKL monoclonal antibody which is used to treat osteoporosis and prevent skeletal damage caused by breast cancer metastases.^1^ RANK (receptor activator of nuclear factor κB) and its ligand (RANKL) are known for their involvement in bone metabolism.^2^ Binding of RANKL to RANK on osteoclast precursors induces osteoclast maturation and activation, thereby stimulating bone resorption. In contrast, binding of RANKL either pharmacologically or by osteoprotegerin (OPG --the endogenous decoy receptor for RANKL) inhibits the RANKL-mediated signaling pathway, consequently inhibiting bone resorption and maintaining bone density. A large trial of over 7000 older women with osteoporosis demonstrated that denosumab significantly reduced the risk of fractures.^3^ Based on those findings, denosumab was added to the Ontario Drug Benefits formulary in 2012 for the treatment of osteoporosis under restricted criteria that requires prior exposure to, or hypersensitivity following oral bisphosphonate use.^4^ It is estimated that 1% of older women in Ontario have initiated this drug annually since its addition to the provincial drug formulary.^5^ The typical schedule for denosumab is one subcutaneous injection every 6 months.

There is emerging evidence that progesterone-mediated up-regulation of the RANK/RANKL also plays a critical role in mammary gland epithelial cell proliferation, in mammary stem cell expansion, and in mammary carcinogenesis.^6–10^ Furthermore, preliminary findings from the adjuvant denosumab in breast cancer (ABCSG 18) double-blind, placebo-controlled trial showed improved disease-free survival among the women randomized to denosumab injected subcutaneously twice a year.^11,12^ The impact of denosumab on the incidence of a second primary cancer has yet to be reported. Targeting of RANK-signaling may be particularly relevant for women at a high risk of developing breast cancer attributed to an inherited mutation in *BRCA1*.^6–10^

The current landscape of chemoprevention for women at high-risk of developing breast cancer consists of either selective estrogen receptor modulators (i.e., SERMs) or aromatase inhibitors and is dependent on menopausal status.^13^ Given the suboptimal uptake of the current prevention options, it is important to identify novel and highly effective therapeutic cancer prevention strategies.^14^ Given the seminal preclinical evidence supporting a role of aberrant RANK-signaling in the development of breast cancer, it is of interest to evaluate whether denosumab with a relatively safe toxicity profile, is a potential candidate.^15^ To our knowledge, there are no studies that have specifically evaluated the relationship between denosumab use and breast cancer risk. The objective of the current study was to utilize large healthcare administrative databases to evaluate the incidence of breast cancer in a large cohort of postmenopausal women following denosumab initiation.

## Materials and Methods

### Study design and data sources

We conducted a population-based matched cohort study using healthcare administrative databases in Ontario, Canada. The databases contain records for all individuals eligible for the province’s universal health coverage. The databases included in this analysis were: the Registered Persons Database files for demographic information (e.g., birth date, death date, sex); the Ontario Cancer Registry (OCR)^16^ to identify invasive breast cancer and cancer history; the Canadian Institute for Health Information Hospital Discharge Abstract Database (CIHI-DAD/) for information regarding hospital admissions; the National Ambulatory Care Reporting System (NACRS) for emergency department visits and day surgeries; the Ontario Drug Benefit (ODB) database for prescription drug claims records as all residents of Ontario aged 65 years and older are eligible for provincial drug coverage through the ODB;^17,18^ and the Ontario Health Insurance Plan (OHIP) for information about physician service claims including mammography history. These datasets were linked using unique encoded identifiers and analyzed at the Institute for Clinical Evaluative Sciences (ICES).

This study was approved by the Research Ethics Board of the Sunnybrook Health Sciences Centre and Women’s College Hospital.

### Study eligibility

Given that eligibility for ODB coverage of denosumab is predominantly restricted to women with a history of oral bisphosphonate use, and breast cancer risk is suggested to be lower among women with osteoporosis and also following bisphosphonate exposure;^19–21^ we restricted inclusion to women with prior oral bisphosphonate use to isolate effects of denosumab. Three oral bisphosphonates are approved for osteoporosis in Canada (alendronate, etidronate, and risedronate), and have been available through the ODB program since 1996.^22^ Bisphosphonate use prior to the date of study entry (i.e., index date as defined below) was obtained for each subject and the cumulative use and time since most recent use was calculated. Women were excluded if they were over the age of 85 at index date, ineligible for OHIP at any point in the two years preceding the index date, had no ODB claims in the two year preceding the index date, had a history of any cancer (excluding non-melanoma skin cancer, ICD-10 any C44) at any point prior to the index date, had a history of any conditions that would impact bone quality at any point prior to the index date (e.g., celiac disease, Cushing’s syndrome, hypercalcemia, hyperparathyroidism, organ transplant, osteomalacia, osteopetrosis, Paget’s disease or renal disease), had a death date preceding/on the index date and had a history of living in a long-term care setting.

### Denosumab exposure

We identified all women aged 67 or more years who received a first prescription of denosumab between 29 February 2012 (first date on ODB formulary) and 30 April 2016. The index date was the first date of dispensation of denosumab. The total number of denosumab prescriptions dispensed at least four months from each other ( ≥ 120 days) were then categorized as 1–2 doses, 3–4 doses, and ≥ 5 doses. All women meeting our inclusion criteria with oral bisphosphonate exposure since 1996 were assigned a random index date based on the distribution of index dates among the eligible exposed subjects.

### Covariates

We also collected information on the following covariates: age, resident location (i.e., rural vs. urban) determined by linking postal codes to census data, income status based on neighborhood income quintile, number of primary care visits, emergency department (ED) visits or acute care hospitalizations in the previous year, screening mammogram in the two years prior to the index date and comorbidity using the John Hopkins aggregated diagnosis groups (ADG) score in the two years prior to the index date,^23^ as well as history of pathologic or other fractures, documentation of osteoporosis, and prior use of other drugs that may impact bone health (i.e., calcitonin, raloxifene).

### Matching

All women aged 67 or more years with oral bisphosphonate exposure since 1996 were assigned a random index date based on the distribution of index dates among the eligible exposed subjects; this ensured that exposed women could serve unexposed time prior to denosumab initiation. We conducted 3:1 matching of unexposed to exposed subjects. Subjects were matched on age ( ± 2 years), index date ( ± 1 year), cumulative use of bisphosphonates ( ± 1 year), time since last use of a bisphosphonate ( ± 1 year) and propensity score. Propensity scores were generated using variables for urban vs. rural residence, historic ED and inpatient visits, ADGs, history of fracture, and historic use of any anti-estrogen therapy, estrogen therapy, or aromatase inhibitors. We conducted propensity score calliper matching using a calliper of 0.2 times the standard deviation of the propensity score.

### Outcomes

The primary outcome of interest was a diagnosis of incident invasive breast cancer documented in OCR in the follow-up period. Incident breast cancers included any malignant neoplasms of the breast (ICD-10 any C50). Benign neoplasms of the breast (ICD-10 any D24), carcinomas in situ of the breast (ICD-10 any D05) and neoplasms of uncertain behavior of the breast (ICD-10 D485 or D486) were not included.

### Statistical analysis

Baseline descriptive characteristics of the two groups were compared using standardized differences. A standardized difference of <0.10 was used to determine comparability between the groups for each covariate of interest.^24^ Cox proportional hazards models, stratified on matched pairs was used to estimate the adjusted hazard ratio (HR) and 95% confidence intervals (CI) for denosumab exposure, as well as increasing dose and time since last bisphosphonate use, and the risk of breast cancer. Women were followed from their index date to either: (1) an incident breast cancer diagnosis, (2) other cancer diagnosis (excluding non-melanoma skin cancer), (3) death, or (4) end of follow-up (31 August 2017), whichever occurred first. Unexposed subjects who eventually received denosumab in the follow-up were also censored (n = 2,201).

All statistical analyses were performed using SAS software version 9.3.

## Results

A total of 100,368 women with a history of bisphosphonate use were included in the final analysis. Of these, 25,092 women were denosumab users and 75,276 were matched non-users. Prior to matching, users and non-users of denosumab differed with respect to age, residential location, bisphosphonate use, history of fall-related injuries, fractures, primary care visits, emergency department visits and acute care hospitalizations in the past year, as well as, total aggregated diagnosis groups. However, following matching for age, index date, bisphosphonate use and propensity score, the two groups of women were similar with respect to all the baseline characteristics (Table 1). The mean number of denosumab doses in the exposed group was 4.8 (standard deviation = 2.9; range 1–12).

**Table 1:** Characteristics of propensity score matched cohort, among all women and by denosumab use

Variable, *n* (%) unless otherwise noted	Value	Total	No denosumab	Denosumab	Standardized difference
		*N* = 100,368	*N* = 75,276	N = 25,092	
Age at index date	Mean ± SD	76.3 ± 4.9	76.3 ± 4.9	76.3 ± 4.9	0.01
	Median (IQR)	76 (72 – 80)	76 (72 – 80)	77 (72 – 80)	0.01
	67 – 69	13,778 (14%)	10,406 (14%)	3372 (13%)	0.01
	70 – 74	26,574 (26%)	20,142 (27%)	6432 (26%)	0.03
	75 – 79	32,714 (33%)	24,353 (32%)	8361 (33%)	0.02
	80 +	27,302 (27%)	20,375 (27%)	6927 (28%)	0.01
Resident location	Urban	93,279 (93%)	70,017 (93%)	23,262 (93%)	0.01
	Rural	7089 (7%)	5259 (7%)	1830 (7%)	0.01
Income quintile	Missing	283 (0%)	224 (0%)	59 (0%)	0.01
	1 – Lowest	18,816 (19%)	14,391 (19%)	4425 (18%)	0.04
	2	21,022 (21%)	15,791 (21%)	5231 (21%)	0
	3	19,808 (20%)	14,919 (20%)	4889 (19%)	0.01
	4	20,839 (21%)	15,542 (21%)	5297 (21%)	0.01
	5 – Highest	19,600 (20%)	14,409 (19%)	5191 (21%)	0.04
Years taking bisphosphonate	Mean ± SD	4.9 ± 3.7	4.9 ± 3.7	4.9 ± 3.7	0
	Median (IQR)	4 (2 – 8)	4 (2 – 8)	4 (2 – 8)	0
	<1 year	17,988 (18%)	13,390 (18%)	4598 (18%)	0.01
	1 – 2 years	20,693 (21%)	15,654 (21%)	5039 (20%)	0.02
	3 – 5 years	25,880 (26%)	19,388 (26%)	6492 (26%)	0
	6 – 9 years	24,454 (24%)	18,354 (24%)	6100 (24%)	0
	10 + years	11,353 (11%)	8490 (11%)	2863 (11%)	0
Years since last bisphosphonate use	Mean ± SD	1.1 ± 2.0	1.1 ± 2.0	1.1 ± 2.0	0.02
	Median (IQR)	0 (0 – 1)	0 (0 – 1)	0 (0 – 1)	0.21
	<1 year	71,959 (72%)	54,211 (72%)	17,748 (71%)	0.03
	1 – 2 years	14,950 (15%)	10,959 (15%)	3991 (16%)	0.04
	3 – 5 years	9402 (9%)	7056 (9%)	2346 (9%)	0
	6 + years	4057 (4%)	3050 (4%)	1007 (4%)	0
Primary care visit(s) in the previous year	Yes	95,732 (95%)	71,441 (95%)	24,291 (97%)	0.1
	Mean ± SD	6.0 ± 5.1	5.9 ± 5.0	6.3 ± 5.2	0.07
	Median (IQR)	5 (3 – 8)	5 (3 – 8)	5 (3 – 8)	0.09
Emergency department visit(s) in the previous year	Yes	27,275 (27%)	20,389 (27%)	6886 (27%)	0.01
	Mean ± SD	0.5 ± 1.1	0.5 ± 1.1	0.5 ± 1.3	0
	Median (IQR)	0 (0 – 1)	0 (0 – 1)	0 (0 – 1)	0.01
Acute care hospitalization(s) in the previous year	Yes	5612 (6%)	4230 (6%)	1382 (6%)	0
	Mean ± SD	0.1 ± 0.3	0.1 ± 0.3	0.1 ± 0.3	0.01
	Median (IQR)	0 (0 – 0)	0 (0 – 0)	0 (0 – 0)	0
Mammogram(s) in the previous 2 years	Yes	39,165 (39%)	28,803 (38%)	10,362 (41%)	0.06
	Mean ± SD	0.5 ± 0.6	0.4 ± 0.6	0.5 ± 0.6	0.07
	Median (IQR)	0 (0 – 1)	0 (0 – 1)	0 (0 – 1)	0.07
	No mammograms	61,203 (61%)	46,473 (62%)	14,730 (59%)	0.06
	1 mammogram	32,353 (32%)	23,925 (32%)	8428 (34%)	0.04
	2 mammograms	6702 (7%)	4807 (6%)	1895 (8%)	0.05
	3+ mammograms	110 (0%)	71 (0%)	39 (0%)	0.02
Aggregated Diagnosis Groups (ADGs) (2 year lookback)	Mean ± SD	7.4 ± 3.3	7.4 ± 3.3	7.5 ± 3.3	0.03
	Median (IQR)	7 (5 – 10)	7 (5 – 10)	7 (5 – 10)	0.02
	0 – 4 ADGs	19,668 (20%)	14,821 (20%)	4847 (19%)	0.01
	5 – 9 ADGs	55,259 (55%)	41,545 (55%)	13,714 (55%)	0.01
	10 + ADGs	25,441 (25%)	18,910 (25%)	6531 (26%)	0.02
Fall-related Injury	Yes	7846 (8%)	5814 (8%)	2032 (8%)	0.01
Nonfall-related injury	Yes	4811 (5%)	3615 (5%)	1196 (5%)	0
Any fracture diagnosis	Yes	4083 (4%)	3026 (4%)	1057 (4%)	0.01
Concurrent medication use (excluding bisphosphonates and denosumab)	Mean ± SD	3.2 ± 2.8	3.2 ± 2.8	3.1 ± 2.7	0.05
	Median (IQR)	3 (1 – 5)	3 (1 – 5)	3 (1 – 5)	0.05
	0 medications	16,966 (17%)	12,701 (17%)	4265 (17%)	0
	1 – 4 medications	55,974 (56%)	41,610 (55%)	14,364 (57%)	0.04
	5 – 9 medications	24,348 (24%)	18,577 (25%)	5771 (23%)	0.04
	10 + medications	3080 (3%)	2388 (3%)	692 (3%)	0.02
Follow up in years	Mean ± SD	2.8 ± 1.5	2.8 ± 1.5	2.8 ± 1.5	0.02
	Median (IQR)	3 (1 – 4)	3 (1 – 4)	3 (1 – 4)	0.02
Breast cancer diagnosis^a^	Yes	1271 (1%)	986 (1%)	285 (1%)	0.02
Other cancer diagnosis^a^	Yes	3022 (3%)	2261 (3%)	761 (3%)	0
Death^a^	Yes	5306 (5%)	3970 (5%)	1336 (5%)	0

^a^Note: censoring not included in these outcomes. Outcomes are based on experiencing the event at any point in follow-up.

**Fig. 1:**
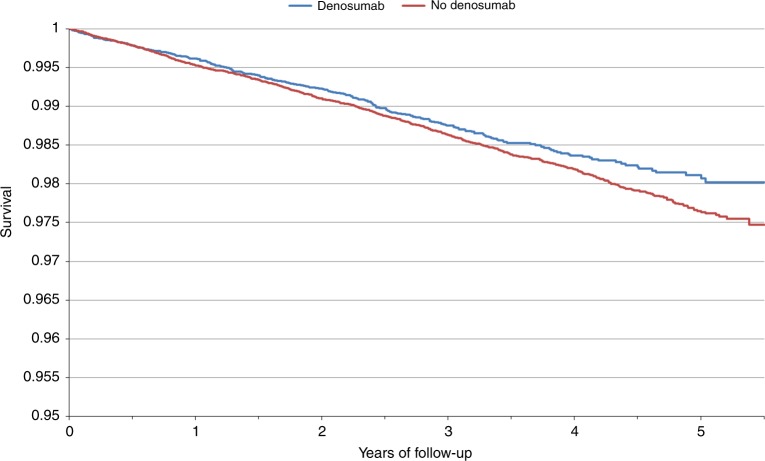
Kaplan-Meier curve of breast cancer-free survival over follow-up time by denosumab use

A total of 1271 women were diagnosed with breast cancer over the follow-up period with 285 (1.1%) cases diagnosed among the denosumab users and 986 (1.3%) cases among the non-users (Table 1). Women were followed for an average of 2.8 years, reflecting an overall protective effect with denosumab exposure (HR = 0.87, 95% CI 0.76–1.00) (Table 2). The 5-year cumulative incidence of breast cancer was 1.9% in denosumab users and 2.4% among the non-users (*P* – log rank = 0.04), Figure 1. The relationship did not vary by increasing cumulative dose (*P* – trend = 0.15) or time since last use of bisphosphonates (*P* – interaction = 0.52) (Table 2).

**Table 2:** The association between denosumab use and breast cancer risk

Strata	BC events		Univariate	
	No denosumab	Denosumab	HR (95% CI)	*P*
Ever/never denosumab use	957	281	0.87 (0.76–1.00)	0.04
Cumulative dose				
Per injection			0.98 (0.94–1.01)	0.15^a^
1–2 injections	-	124	0.82 (0.68–1.00)	0.04
3–4 injections	-	71	0.88 (0.69–1.13)	0.32
5+injections	-	86	0.95 (0.75–1.20)	0.65

^a^P – Trend

The characteristics of the breast cancer patients are summarized in Table 3 by history of denosumab use. On average, women in the denosumab group had slightly more nodal involvement compared to women in the no denosumab group (mean nodal involvement 1.1 vs. 0.6); however, this was only different for the continuous variable (number of nodes) and not the type of nodal involvement (positive or negative). The two groups were similar with respect to various factors including stage, size, grade, hormone-receptor status or HER2 status of the incident breast cancers.

**Table 3:** Breast cancer characteristics of all women and by denosumab use

Variable, *n* (%) unless otherwise noted	Value	Total	No denosumab	Denosumab	*P*
		*N*=1238 (%)	*N*=957 (%)	*N*=281 (%)	
Diagnosis year	2012	47 (4)	37 (4)	10 (4)	0.14
	2013	117 (9)	81 (8)	36 (13)	
	2014	177 (14)	139 (15)	38 (14)	
	2015	263 (21)	205 (21)	58 (21)	
	2016	376 (30)	284 (30)	92 (33)	
	2017	258 (21)	211 (22)	47 (17)	
Stage	Unknown	307 (25)	242 (25)	65 (23)	
	I	405 (44)	316 (44)	89 (41)	0.28
	II	350 (38)	267 (37)	83 (38)	
	III	113 (12)	80 (11)	33 (15)	
	IV	63 (7)	52 (7)	11 (5)	
Tumor size (continuous) (cm)	Mean±SD	2.6±2.0	2.6±2.0	2.7±2.0	0.50
	Median (IQR)	2 (1–3)	2 (1–3)	2 (1–4)	0.45
Tumor size (categorical) (cm)	Unknown	532 (43)	428 (45)	104 (37)	
	<1 cm	103 (15)	75 (14)	28 (16)	0.57
	1–2 cm	222 (31)	174 (33)	48 (27)	
	2–3 cm	161 (23)	120 (23)	41 (23)	
	3–5 cm	145 (21)	108 (20)	37 (21)	
	5+ cm	75 (11)	52 (10)	23 (13)	
Nodes involved (continuous)	Mean±SD	0.7±2.2	0.6±1.6	1.1±3.3	0.03
	Median (IQR)	0 (0–1)	0 (0–1)	0 (0–1)	0.37
Nodes involved (categorical)	Unknown	640 (52)	509 (53)	131 (47)	
	Node negative	421 (70)	317 (71)	104 (69)	0.74
	Node positive	177 (30)	131 (29)	46 (31)	
Grade	Unknown	651 (53)	522 (55)	129 (46)	
	Low grade	126 (21)	94 (22)	32 (21)	0.98
	Medium grade	306 (52)	227 (52)	79 (52)	
	High grade	155 (26)	114 (26)	41 (27)	
ER status	Unknown	574 (46)	462 (48)	112 (40)	
	Negative	89 (13)	64 (13)	25 (15)	0.54
	Positive	575 (87)	431 (87)	144 (85)	
PR status	Unknown	582 (47)	467 (49)	115 (41)	
	Negative	158 (24)	117 (24)	41 (25)	0.83
	Positive	498 (76)	373 (76)	125 (75)	
HER2 status	Unknown	602 (49)	477 (50)	125 (44)	
	Borderline	165 (26)	127 (26)	38 (24)	0.62
	Negative	417 (66)	315 (66)	102 (65)	
	Positive	54 (8)	38 (8)	16 (10)	

In a sensitivity analysis, the inclusion cohort was re-matched such that the index date for denosumab users was 6 months following their initial injection to allow for a 6 month lag period between denosumb use and breast cancer risk. The findings did not change considerably. For example, the HR comparing users vs. non-users was 0.86 (95% CI 0.74–0.99; *P* = 0.04)(data not shown).

## Discussion

In this large, population-based study of older women with a history of oral bisphosphonate exposure we observed that use of denosumab was associated with a modestly significant 13% decreased risk of subsequent breast cancer. After 5 years of follow-up, the cumulative incidence of breast cancer was significantly lower in the denosumab users vs. the non-users (1.9% vs. 2.4%). There was no dose-response relationship and the association did not vary by time since last bisphosphonate use. Except for nodal involvement, pathologic, and histologic characteristics of the breast tumors did not vary by history of denosumab exposure. Although these findings suggest a protective effect of denosumab exposure, they should be interpreted with caution given the relatively short duration of follow-up in this analysis as well as the median age of the population (~76 years). It is of interest to confirm our findings in other studies and to further evaluate the relationship between denosumab and breast cancer in younger patients and with additional years of follow-up.

Our cohort consisted of women from the general population with a history of bisphosphonate use, and thus, are not representative of women in the general population. Although we found little evidence of a dose-response association between denosumab exposure and subsequent breast cancer risk, our findings with any denosumab use do not preclude a potential chemoprevention role of this drug among women without a history of osteoporosis including those under the age of 67. Indeed, our results suggests that a short-course of denosumab has the potential to offer long-term protection against breast cancer which is analogous to the cancer protective effects conferred by a later age at menarche and breastfeeding, intrinsic, and transient exposures that significantly reduce the risk of breast cancer.^25^ On the other hand, we cannot rule-out some residual confounding related to denosumab initiation. For example, women treated with denosumab (a new drug to market) may be healthier in terms of diet, alcohol consumption and exercise – aware of the new pharmacological option and broaching the discussion with their physician, or treated by attentive physicians encouraging preventive health behaviors.

Despite the inclusion of an older population in the current study, one cannot preclude a potential breast cancer protective effect of denosumab (or other RANKL inhibitors) in younger high-risk populations, particularly among women with an inherited *BRCA1* mutation. Pre-clinical findings from various seminal publications have collectively elucidated a pivotal role of the RANK-pathway in *brca1* mammary carcinogenesis.^6,8,10,26,27^ Specifically, Nolan et al., demonstrated that RANKL inhibition resulted in a significant delay in mammary tumor onset and incidence in a *brca1* deficient mouse model, and furthermore, that treatment of premenopausal women with denosumab resulted in a substantial reduction in breast epithelial cellular proliferation based on Ki67 expression^27^ and confirmed by an independent research group.^26^ These findings are of particular relevance for women with a *BRCA1* mutation given their high lifetime risk of developing breast cancer, the very limited data regarding tamoxifen use for primary prevention, along with the suboptimal uptake of tamoxifen since most *BRCA* mutation carriers opt for yearly screening with MRI.^28^ Randomized trials or observational intervention trials in this specific population are highly anticipated. We did not have information on family history or *BRCA* mutation status, and thus, were not able to assess risk in these subgroups.

The prevention and treatment of postmenopausal osteoporosis have historically included the use of bisphosphonates, a class of drugs that inhibits osteoclast-mediated bone resorption.^29^ Intravenous bisphosphonates are also prescribed to breast (and other) cancer patients to prevent treatment-induced skeletal complications including bone loss and bone metastases.^30^ Evidence from earlier, epidemiologic studies suggested a possible reduction in breast cancer risk among postmenopausal women who used bisphosphonates;^20,21^ however, a results from a recent prospective cohort of 64,438 French postmenopausal women and 2407 incident cases, reported no significant association between bisphosphonate use and breast cancer risk (HR = 0.98, 95% CI 0.85–1.12).^31^

There are several limitations to our study. First, the duration of follow-up was short (on average ~2.8 years). This was purely attributed to the fairly recent introduction of denosumab for the treatment of osteoporosis. We wanted to ensure accurate information regarding prescribed denosumab use, and thus, only included women who were 67 years of age or older who had at least 2 prior years of coverage under the ODB program. However, it should be noted that denosumab for the treatment of osteoporosis was not added to the Ontario provincial formulary until February 2012 and is only provided in special circumstances. This may have resulted in some misclassification including under-capturing if patients received drug coverage through other mechanisms (e.g., out-of-pocket, private insurance).^32^ Given that more than half of the breast cancers in Canada are diagnosed prior to age 69, we did not capture the full population of interest. The women included in the current analysis were limited to older (on average 76 years of age) women with a history of bisphosphonate use, and likely not representative of the larger number of women at risk of developing breast cancer. Although we did not have information on various breast cancer risk factors including family history, we were able to demonstrate that both groups of women were similar with respect to screening (Table 1) and use of chemopreventive drugs such as raloxifene and tamoxifen (data not shown). Furthermore, reproductive and hormonal risk factors are unlikely to differ based on initiation of denosumab.

Despite these limitations, our study had several strengths, in particular, the use of large provincial administrative datasets, allowing for well-powered analyses and matching on relevant confounders. Our exposed and unexposed groups were similar with respect to most demographic characteristics, prior history of bisphosphonate use and other medications that may impact bone health (e.g., estrogen therapy, calcitonin), comorbidities, as well as, health care utilization patterns. Comprehensive ODB drug data permits capture of oral bisphosphonate therapy since 1996, matching on age and calendar time helps control for changes in osteoporosis management and therapy over time.

In conclusion, we found a small inverse relationship between denosumab exposure and breast cancer incidence in this large population-based study of older women residing in Ontario, Canada. To our knowledge, this represents the first report of denosumab use and subsequent breast cancer risk. Further studies with a longer follow-up period, as well as the inclusion of younger women or cohorts of high-risk women, are necessary to delineate the role of RANK-inhibition in the prevention of breast cancer. It is of importance to establish whether denosumab, administered subcutaneously as a semi-annual injection with a safe toxicity profile, has the potential to be used in the primary prevention setting.

## Data Availability

The data analyzed for the current study are available upon reasonable request from the corresponding author.
